# Human olfactory mesenchymal stromal cell transplants promote remyelination and earlier improvement in gait co‐ordination after spinal cord injury

**DOI:** 10.1002/glia.23117

**Published:** 2017-02-01

**Authors:** Susan L. Lindsay, Andrew Toft, Jacob Griffin, Ahmed M. M. Emraja, Susan Carol Barnett, John S. Riddell

**Affiliations:** ^1^ Institute of InfectionCollege of Medical Veterinary and Life Sciences, Inflammation, and Immunity, University of GlasgowGlasgowG12 8TAUnited Kingdom; ^2^ Institute of Neuroscience and PsychologyCollege of Medical Veterinary and Life Sciences, West Medical Building, University of GlasgowG12 8QQUnited Kingdom

**Keywords:** spinal cord injury, transplant, mesenchymal stromal cells, remyelination, gait analysis

## Abstract

Autologous cell transplantation is a promising strategy for repair of the injured spinal cord. Here we have studied the repair potential of mesenchymal stromal cells isolated from the human olfactory mucosa after transplantation into a rodent model of incomplete spinal cord injury. Investigation of peripheral type remyelination at the injury site using immunocytochemistry for P0, showed a more extensive distribution in transplanted compared with control animals. In addition to the typical distribution in the dorsal columns (common to all animals), in transplanted animals only, P0 immunolabelling was consistently detected in white matter lateral and ventral to the injury site. Transplanted animals also showed reduced cavitation. Several functional outcome measures including end‐point electrophysiological testing of dorsal column conduction and weekly behavioural testing of BBB, weight bearing and pain, showed no difference between transplanted and control animals. However, gait analysis revealed an earlier recovery of co‐ordination between forelimb and hindlimb stepping in transplanted animals. This improvement in gait may be associated with the enhanced myelination in ventral and lateral white matter, where fibre tracts important for locomotion reside. Autologous transplantation of mesenchymal stromal cells from the olfactory mucosa may therefore be therapeutically beneficial in the treatment of spinal cord injury. GLIA 2017 GLIA 2017;65:639–656

## Introduction

Spinal cord injury (SCI) is a devastating condition that can lead to severe functional deficits which are usually permanent because of the limited capacity of the adult central nervous system (CNS) for repair. One promising strategy for therapy is cell transplantation which has reached the stage of clinical translation. The olfactory system has been a source of cells for such studies (Feron et al., 2005; Lima et al., 2010, 2006; Lu et al., 2002; Mackay‐Sim et al., 2008) because of the presence of specialised glia called olfactory ensheathing cells (OECs), with properties considered conducive to CNS regeneration (Barnett et al., 1993; Mackay‐Sim et al., 2008; Raisman, 2007; Ramer et al., 2004; Ramon‐Cueto and Nieto‐Sampedro, 1994). In addition, the olfactory mucosa offers a relatively accessible source of cells for autologous transplantation, which avoids the need for immunosuppression (Feron et al., 2005).

Graft composition has varied in different studies using olfactory tissue. Some preclinical studies have investigated purified populations of OECs (Ramer et al., 2004; Ramon‐Cueto and Nieto‐Sampedro, 1994; Richter et al., 2005; Riddell et al., 2004; Toft et al., 2007) while many animal studies, and the majority of clinical studies, have investigated mixtures of cell types (Chhabra et al., 2009; Lima et al., 2010, 2006). These have included OECs mixed with fibroblast‐like cells (Choi et al., 2008; Feron et al., 2005; Tabakow et al., 2013), and undissociated pieces of mucosa (Chhabra et al., 2009; Lima et al., 2010, 2006). However, the precise cell types grafted into patients have rarely been well characterised so that although clinical studies suggest that the procedure of isolating olfactory mucosa and transplantation itself are mostly safe (Chhabra et al., 2009; Feron et al., 2005; Lima et al., 2010, 2006), the optimum cell types for promoting repair remain unclear.

We have purified mesenchymal‐like stromal cells (MSCs) from the lamina propria of human olfactory mucosa which we have termed hOM‐MSCs (Lindsay et al., 2013). We have demonstrated that conditioned media from these cells causes oligodendrocyte precursor cells (OPCs) to proliferate and extend processes, and importantly that it promotes CNS myelination *in vitro* (Lindsay et al., 2013). MSCs have been extensively investigated for transplant mediated repair of SCI. However, attention has been focussed on MSCs derived from the bone marrow (BM‐MSCs) since these cells can be easily and reproducibly isolated from aspirates and transplanted as autografts. Preclinical studies suggest that MSCs reduce tissue damage, decrease cyst and injury size, and modestly improve functional outcomes in some models of SCI (Chopp et al., 2000; Himes et al., 2006; Neuhuber et al., 2005). However, enhanced remyelination has not been reported and consistent with this, conditioned media from BM‐MSCs does not promote myelination *in vitro* (Lindsay et al., 2013). MSCs obtained from the olfactory mucosa therefore represent an additional source of cells which could be used in an autologous transplantation strategy for SCI repair. They may be easier to obtain and expand in culture to the volumes required for transplantation into human spinal cord injuries than other olfactory mucosa cell types, and may have additional repair properties, such as the promotion of myelination (Lindsay et al., 2013). We have therefore investigated the repair properties of hOM‐MSCs in a rodent model of SCI. We found that although there was no long‐term enhancement of dorsal column long tract function, there was an earlier recovery of co‐ordinated stepping in transplanted animals and this was correlated with enhanced peripheral type remyelination of fibres in areas of white matter known to be important for locomotion.

## Materials and Methods

### Experimental Design

**Figure 1:**
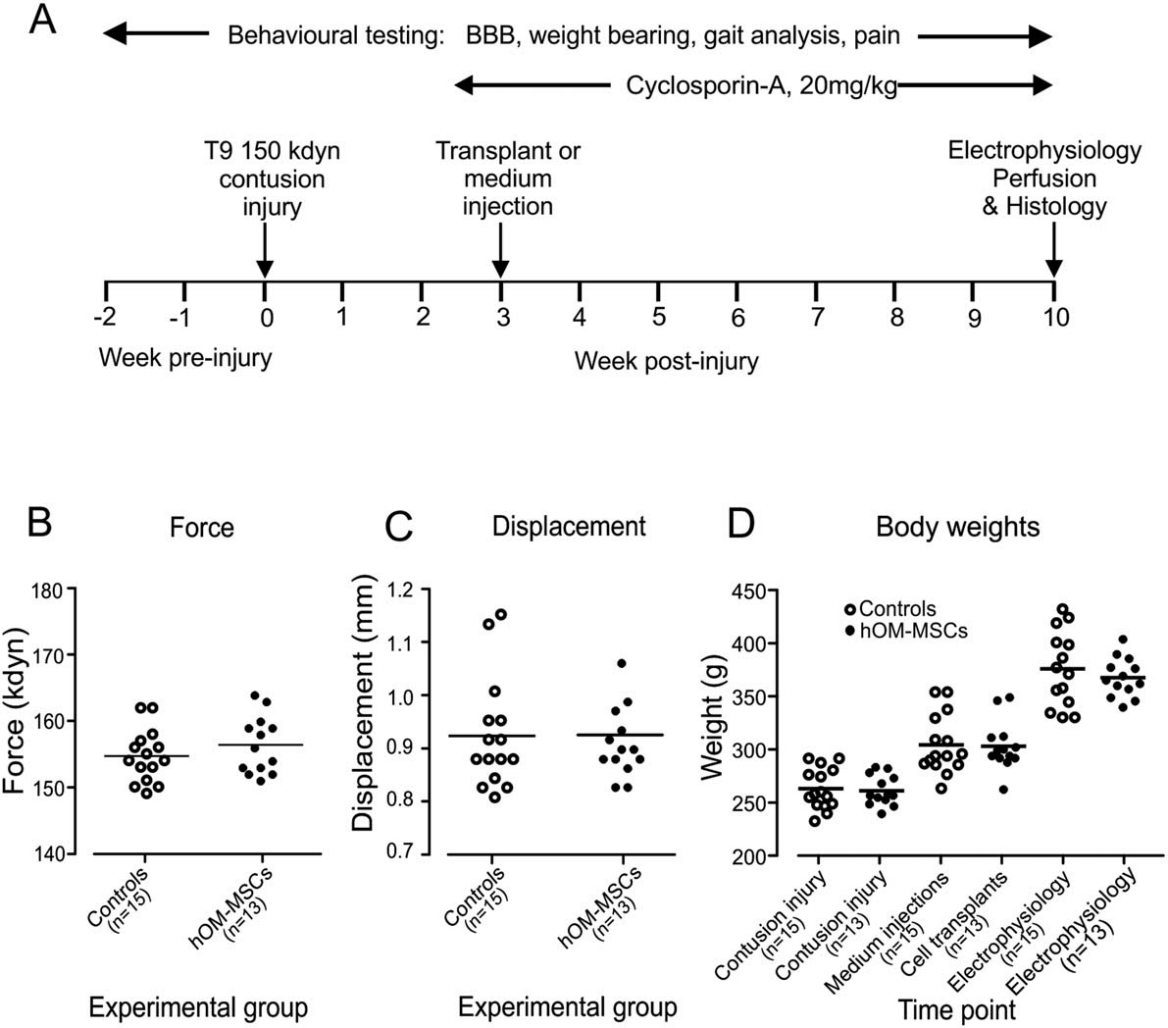
Experimental design and injury parameters. (**A**) Schematic diagram summarizing the experimental time‐line. (**B**) Injury force and (**C**) displacement were similarly distributed for transplant and control groups and showed no significant difference (Student's *t* test). (**D**) Body weights were similarly distributed within each group throughout the study with no significant differences (Student's unpaired *t* test). In plots B–C horizontal bars represent mean values.

Figure 1A summarises the experimental design. Animals were subjected to low thoracic contusion injuries and after 3 weeks, were either transplanted with cells or injected with media. All animals were started on daily immunosuppression prior to transplantation/media injection, which continued for the study duration. Functional outcome was assessed using behavioural and electrophysiological tests. Acclimatisation started 2 weeks before injury and behavioural testing was performed prior to injury and at regular intervals for 9 weeks thereafter. Electrophysiological testing was performed at the end of the experiment and animals perfusion fixed for anatomical analysis. Animals were set up in batches and randomly assigned to transplant or control groups at the time of injury with similar numbers of animals in each group. Behavioural tests and analysis of results were carried out blind. Sample size calculations were performed using a conventional protocol as previously detailed (Chow et al., 2008). This calculation assumed that there were no differences in standard deviations between groups and that detection of 20% change is derived with 80% power at a 5% level of significance, for two‐sided significance tests. Animal numbers were set up accordingly to ensure that for each outcome measure the group size from which data was obtained was appropriate (*n* = 10–15 control or transplanted animals, see *Animals* and Table 1).

**Table 1:** Batch Information for Donor Cells, Animal Numbers, and Outcome Measures

	**Fold increase for *in vitro* myelination**	**Total number of animals**		**Number of animals for electro‐ physiology and sensorimotortests**		**Number of animals for gait analysis**		**Number of animals assessed for P0**	
		Cells	Media	Cells	Media	Cells	Media	Cells	Media
Donor 1/Batch 1	1.83	4	3	3	3	2	1	0	0
Donor 2/Batch 2	1.80	5	5	5	5	3	5	1	1
Donor 3/Batch 3	2.46	5	7	5	7	4	4	2	2
Donor 4/Batch 4	2.01	1	4	0	0	0	0	1	4
**Total**		**15**	**19**	**13**	**15**	**9**	**10**	**4**	**7**

Table shows the degree of *in vitro* myelination produced by conditioned media collected from hOM‐MSCs obtained from each donor. Myelination is shown as a fold increase relative to control cultures and is similar to that reported previously (Lindsay *et al*., 2013). The number of animals transplanted with cells from each donor and the corresponding media injected animals set up within each batch are shown. Table also details the number of animals from which sensorimotor and electrophysiological data was obtained and the final number of animals that were included for gait analysis (animals which could not complete one or more tests were omitted). Final column shows the numbers of animals used for analysis of P0 immunolabelling.

### Human Olfactory Mucosa Biopsies

Four olfactory mucosa biopsies were obtained with WoSRES approval (07/s0710/24) and informed patient consent from two males, one female, and one patient of undisclosed sex undergoing nasal septoplasty/polypectomy surgery (Table 1). Patients were aged 40, 43, 50, and 53 (average age 46.5 years). Biopsies were taken from superior regions known to contain olfactory mucosa (Morrison and Costanzo, 1990). Biopsies were collected, purified and grown as previously described (Lindsay et al., 2013). After purification, cells (termed hOM‐MSCs) were lentivirally infected using a MOI of 10 (Amsbio UK, LVP001) to produce GFP expressing cells. Lentiviral GFP infection was >98% in all preparations. Conditioned media was harvested from each donor' cells and independently assessed for its potential to promote *in vitro* CNS myelination using previously described methods (Lindsay et al., 2013). Media from all donors was shown to enhance myelination (Table 1).

### Animals

A total of thirty‐seven adult male Sprague Dawley rats were used in the study (200–250 g; Harlan Laboratories, Loughborough, UK). Table 1 details how animals were distributed throughout the study. Twenty‐nine were set up for the behavioural study. One animal failed to recover normally from the contusion injury and was therefore removed. An additional three rats were used for investigation of cell survival and distribution at early time points (10 days and 4 weeks). Five further rats were set up specifically to assess P0 localisation throughout the cord. Animals were housed under a 12 h light/dark cycle with *ad libitum* access to food and water. All experimental procedures were performed in accordance with the UK Animals (Scientific Procedures) Act 1986.

### Contusion Injury Surgery and Postoperative Care

Contusion injuries were performed on thirty‐seven animals using an Infinite Horizon impactor (Precision Systems Instrumentation). Animals were anesthetised with isoflurane and a laminectomy performed to expose the spinal cord at the T9 segmental level. The vertebral column was stabilized using Adson forceps and a midline impact delivered (force 150 kdyn). A 10‐0 ethicon suture was placed in the dura to facilitate identification of the injury site at subsequent transplantation/media injection surgery. The wound was closed and animals recovered in warmed cabinets overnight. Animals received analgesia (buprenorphine, 0.05 mg kg^−1^ and carprofen, 5 mg kg^−1^, s.c., at induction of anaesthesia and the morning after surgery). Saline (3–5 mL) and enrofloxacin (5 mg kg^−1^) were given s.c. for 3 and 7 days, respectively, after injury. Bladders were manually expressed twice daily until reflexive emptying returned (typically 6–7 days after injury).

### Cell Transplantation

Cell/media injection was performed 3 weeks post‐injury. All animals received immunosuppression (cyclosporine, 20 mg kg^−1^, daily, s.c. Novartis, UK) from 2 days before surgery, until the end of the study. Prior to surgery, cells were detached using 0.25% trypsin‐EDTA (Sigma, UK), passed through a 40 μm cell strainer and centrifuged. Pelleted cells were re‐suspended in 50 μL αMEM:10%:EDTA (10% serum:2 mM EDTA) and kept on ice. Animals were anaesthetised with isoflurane and the injury site re‐exposed. A glass micropipette (internal diameter 50–70 μm) was loaded with cells (∼100,000–200,000 cells μL^−1^). The pipette was inserted into the lesion through a slit in the dura to a depth of 1200 μm. Cells were injected by brief (40 ms) pressure pulses (Picoinjector, WPI, Sarasota FL), as the pipette was slowly raised to around 200 μm or until cells overflowed (typically 30–40 μL/animal). Media control animals were injected with similar volumes of αMEM:10%:EDTA. Wounds were closed and animals recovered in a warm environment.

### Basso, Beattie, and Bresnahan Locomotor Scoring

Open field locomotor function was assessed using the Basso, Beattie, and Bresnahan (BBB) locomotor rating scale (Basso *et al*., 1995) and a subscore (Lankhorst and Hamers, 1999). Animals were placed in a circular pen (diameter of 1.5 m) and both hindlimbs were assessed over 5 min. An average of the scores for both limbs was determined for each animal at 5 days after injury and weekly thereafter for 10 weeks.

### DigiGait™ Analysis

Gait analysis was performed using the DigiGait™ system (Mouse Specifics; Quincy, MA). Animals were placed in a Plexi‐glass chamber on a transparent treadmill and videoed from beneath using a high speed camera (80 Hz). Rats were acclimatized to run at 22 cm sec^−1^. At least five, 2‐ to 3‐sec long runs were collected at each test. Testing was performed pre‐injury and weekly from 2 weeks post‐injury until the end of the study. Nine animals did not complete sufficient runs to allow reliable data to be obtained at all post‐injury test sessions. This was mainly due to reduced motivation rather than physical inability to run, since most of the animals (6/9) ran at the first post‐injury test. The loss of animals was similar in transplanted and medium injected groups (three per group). These animals were completely removed from the analysis since their inclusion would have precluded proper statistical analysis. Final gait data was therefore obtained from 9 transplanted and 10 control animals, all of which ran in every test session (summarised in Table 1). Video files were individually reviewed and any steps that were erroneously represented as being in contact with the treadmill were corrected. Videos containing errors which could not be corrected were rejected. Corrected videos were analyzed to obtain a range of gait parameters (Dorman et al., 2014).

### Sensory Testing

Tests of changes in tactile sensitivity were performed on the forepaws (avoiding injury induced hyperreflexia of the hindpaws) and over the back.

#### Forepaw plantar von frey testing

Forepaw withdrawal thresholds to von Frey hairs were determined using the up down method (Dixon, 1980). Rats were placed in a plexiglass chamber set over a mesh platform and tested after 15‐min habituation. Filaments were applied to the plantar surface of a forepaw when animals were stationary, until bending occurred and then pressure maintained for ∼2 sec. A 50% withdrawal thresholds were determined (Chaplan et al., 1994) for each paw on three occasions (5 min between tests) and an average threshold for each paw calculated.

#### Girdle von Frey hair testing

Development of at level pain in response to mechanical stimulation was tested using a 2.44g von Frey hair applied to the animal's back 2 cm caudal and 2 cm lateral to the injury site. Animals were acclimatised in an empty cage for 5–10 min. The von Frey filament was applied for 2–3 sec and held against the skin so that the filament bent. This was repeated 10 times on each side of the animal, with at least 30 sec between applications. The number of applications resulting in one of the following avoidance responses was recorded and averaged for each side: head turning, biting, avoidance (whole body movement) or jumping/flinching. Results for each side were averaged.

### Dynamic Weight Bearing

The distribution of weight between paws was measured using a Dynamic weight bearing device (BIOSEB, France). Animals were placed in a Plexiglas chamber with an array of pressure transducers covering the floor. Information on the force generated by each paw contact was recorded (sampling frequency 10 Hz) together with a synchronised video image of the animal. Off‐line analysis was performed semi‐automatically after operator confirmation of correct paw identification from the video. Animals were tested at 6 weeks post‐injury for a period of 5 min each.

### Electrophysiology

To assess the function of ascending pathways projecting along the dorsal columns, sensory evoked potentials (SEPs) were recorded in acute experiments. Methods were similar to those described previously (Toft et al., 2007). Animals were anaesthetized, with 5% isoflurane and subsequently with doses of sodium pentobarbital (10 mg kg^−1^ i.v.). During recording, when the animals were paralyzed with pancuronium (Sigma, UK; 0.1 mg i.v. at 40‐min intervals) and artificially ventilated, anaesthetic was given at a frequency commensurate with that required before paralysis and monitored by continuously recording blood pressure and its response to noxious stimuli and observing pupil diameter. Core temperature, blood pressure, and end‐tidal pCO_2_ were maintained within physiological limits. The left radial and sciatic nerves were dissected for electrical stimulation and a small laminectomy was performed at the lumbar level to allow recording of the sciatic afferent volley. The animal was stabilised in a frame and a craniotomy performed to expose the right sensorimotor cortex. The dura over the brain and spinal cord was opened and exposed tissues covered with warmed liquid paraffin.

A diagrammatic representation of the electrophysiological recording set up is shown in Fig. 4A and example SEP recordings in Fig. 4B. SEPs were recorded using a monopolar silver‐ball electrode placed on the surface of the sensorimotor cortex while supramaximal shocks (0.5 Hz, 0.2 ms, <500 µA) were applied alternately to radial and sciatic nerves. Recordings with 1 mm spacing were made according to a grid extending 4‐mm rostral and caudal to Bregma and up to 5 mm laterally (see Fig. 4C,D). Up to 40–60 records (without filtering, 20 kHz sampling rate) were averaged using a digital interface and Signal software (Cambridge Electronic Design, Cambridge, UK). Latencies and amplitudes of SEPs were measured and isopotential plots created using 3D field contour plotting software (Version 3.0.9.0, Copyright 1998–2007, Vladimir Galouchko).

### Histology

#### Tissue preparation and immunohistochemistry

Animals were deeply anaesthetized with intraperitoneal sodium pentobarbital (Euthatal, Vericore, UK; 200 mg mL^−1^) and perfused with mammalian Ringer's solution (containing 0.1% lidocaine) followed by depolymerized 4% paraformaldehyde in 0.1 M PB, pH 7.4. A block of tissue encompassing the lesion/transplant or injection sites was removed and post‐fixed overnight in PFA containing 30% sucrose. Sixty micrometer sagittal sections encompassing the injury site were cut on a cryostat and incubated free‐floating for 30 min in 50% ethanol then washed for 10 min in 0.3 M phosphate‐buffered saline (PBS). Sections were then incubated for 72 h at 4°C in different combinations of the following primary antibodies: sheep or chicken anti‐GFP (Abcam; 1:1,000), mouse anti‐GFAP (Sigma, UK; 1:1,000), rabbit anti‐GFAP (DAKO; 1:1,000), mouse/rabbit anti‐NF200 (1:1,000, Sigma), chicken anti‐P0 (1:100 Santa‐Cruz), rabbit anti‐laminin (Sigma, 1:400), rabbit anti‐Caspr (Abcam, 1:500). Sections were subsequently incubated in fluorophore‐conjugated species‐specific donkey IgG secondary antibodies for 4 h at room temperature. Secondary antibodies were conjugated to Alexa‐488 (1:500), Alexa‐647 (1:500) or Rhodamine Red (1:200), all from Jackson ImmunoResearch Laboratories. In four colour reactions, Caspr was revealed using anti‐rabbit biotinylated secondary antibody (1:500, Jackson ImmunoResearch Laboratories) followed by avidin‐Pacific Blue (1:100, Invitrogen). All antibodies were diluted in PBS with 0.3% triton X‐100. Sections were mounted onto plain glass slides using VectaShield (Vector laboratories) and stored at −20°C.

#### Microscopy

Sections were viewed using an epifluorescence microscope (Zeiss, Axioplan) and illustrative material scanned using a confocal microscope (Zeiss LSM 710). Projections of tiled scans were formed from low power (X20, 11–13 optical sections at 2 μm z‐separation) or high power (40× or 63× oil immersion between 9 and 13 optical sections at 0.5 or 0.3 μm z‐separation) scans.

#### Measurement of injury site width and length

Sagittal sections from each injury block were viewed under an epifluorescence microscope and rostral and caudal limits of the injury identified from cavity rims or the boundary between extracellular matrix and glial scar. Four sections containing the most extensive sites were selected for confocal imaging and the maximal injury length measured using ZEN lite 2010 software (Zeiss). Injury site width was quantified by counting the number of sections containing the injury and multiplying by section thickness.

### Semi‐Quantification of Protein‐Zero Immunohistochemistry

All sections from the entire width of the injury site tissue block of media injected and hOM‐MSC transplanted animals used for assessment of peripheral type remyelination were reacted for P0. All sections were then examined by epifluorescence microscopy and P0 expression noted (Fig. 6A). Each section was scored in a categorical manner that is, scored positive or negative for the presence or absence of P0 immunolabelling, irrespective of the amount of immunolabelling. This scoring was performed for three regions of the spared white matter surrounding the injury site: (1) dorsal columns, (2) lateral funiculi and (3) ventral funiculi as shown in Fig. 6A. The number of sections in which P0 immunolabelling was scored as positive was then expressed as a percentage of all sections. This categorical data was then statistically analyzed using Fisher's exact test with *P* < 0.05 taken to be significant. Because there was no significant difference between the numbers of sections obtained from transplanted and media control animals (Student's *t* test), this approach was not affected by differences in the widths of cords.

### Statistical Analysis

Numerical data were statistically analyzed using Prism software version 5.0 (Graphpad Software, USA). All parametric data is presented as the mean ± SEM and analyzed using Student's *t* test or ANOVA with Bonferroni's post‐test adjustment for multiple comparisons where appropriate. Differences were considered significant at *P* < 0.05. Recovery of gait was analyzed using a summary measures approach for weekly repeated measurements on each animal. For each individual animal, measurements of gait symmetry, stride length and stride frequency for weeks 4–8 (period of accelerated recovery after cell transplantation) were analyzed in Minitab using linear regression analysis to generate a linear coefficient value. The linear coefficient values were compared using the Mann‐Whitney test to assess the equality of the slopes between control and transplanted groups for each gait parameter (Matthews, 1993; Vossoughi et al., 2012).

## Results

### Injury and Transplantation

Because *in vitro* findings suggest remyelination is a potential repair mechanism of hOM‐MSCs, we used a contusion injury model known to result in demyelination of spared fibres (James et al., 2011; Totoiu and Keirstead, 2005). A T9 level injury was chosen to allow outcome measures focused on functions involving long white matter tracts and a force of 150 kdyn selected based on its suitability for the proposed functional assessments. The actual force and displacement produced at injury did not differ between the groups of animals subsequently transplanted or injected with media (Fig. 1B,C). Furthermore, body weights of animals did not differ between transplanted or media injected animals pre‐ or post‐injury, or at the time of cell transplantation/media injection (Fig. 1D). Transplants of cells or injections of media were made 3 weeks after injury. Similar numbers of animals were transplanted with cells from each of the three donors and animals were set up in batches in which transplanted and controlled animals were studied side by side. Each batch of cells was verified to promote CNS myelination *in vitro* (Table 1).

### BBB Open Field Locomotor Score

**Figure 2:**
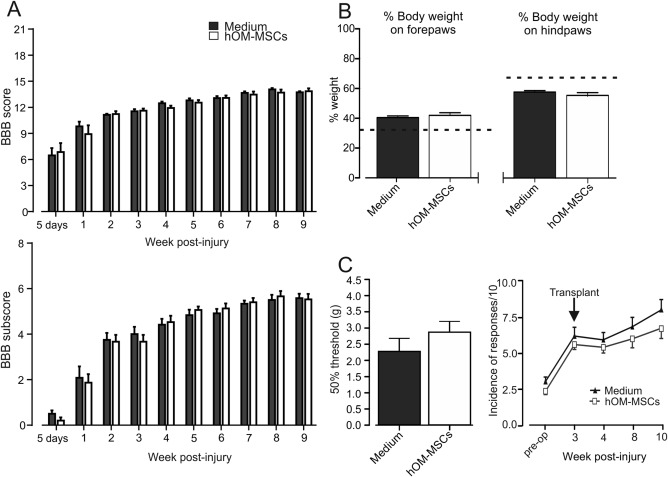
Assessment of long tract functionality. (**A**) BBB locomotor scores and subscores for hOM‐MSC transplanted and media injected animals 5 days post‐operatively and weekly intervals thereafter. Media injected animals, *n* = 15; hOM‐MSC transplanted animals, *n* = 13 (mean ± SEM). There is no significant difference between groups, for either score, at any time point (Repeated measures two‐way ANOVA with Bonferroni's multiple comparison). (**B**) Distribution of body weight between paws 6 weeks after treatment. Mean + SEM, dotted lines indicate normal weight distribution values obtained from un‐operated animals (Emraja and Riddell, unpublished observations). There was no significant difference between groups (Student's unpaired *t* test). (**C**) Assessment of forepaw tactile allodynia 10 weeks after injury using 50% withdrawal thresholds to Von Frey filaments (mean + SEM). hOM‐MSC, *n* = 13; media control *n* = 15. There was a trend towards less allodynia (less lowering in threshold) in transplanted animals however not significant (Student's *t* test). Assessment of tactile allodynia over the back at level. Response to Von Frey hair (0.04 g) applied to skin 2‐cm caudal and 2‐cm lateral to the injury (Mean ± SEM). Animals in both groups developed similar levels of allodynia by 3 weeks after injury. Transplanted animals showed a trend towards less sensitivity, however not significant (Repeated measures two‐way ANOVA with Bonferroni's multiple comparison).

Locomotor recovery was assessed using the BBB open field score and by a subscore method designed to overcome anomalies that can affect the upper end of this scale when used with milder injuries (Lankhorst and Hamers, 1999); the results are shown in Fig. 2A. Five days after injury the mean BBB score was just above 6. This improved progressively over the remainder of the study with the majority of improvement occurring within the first 3 weeks. The second operation to transplant cells or inject media at week 3 post‐injury had little effect and there were also no differences at any later time point.

### Weight Bearing

To assess postural changes, a dynamic weight bearing test was used to measure the percentage of body weight born on each paw. Figure 2B shows the distribution of weight for transplanted and medium injected groups tested 6 weeks after transplantation. In normal animals, when all four paws are in contact with the floor, animals bear more weight on the hindpaws than forepaws (dashed lines in Fig. 2B). Following injury there is a redistribution of weight from the hindpaws to the forepaws. This re‐distribution does not differ between transplanted and control animals so that transplants do not alter this postural change.

### Pain Assessment

The effect of hOM‐MSC transplants on post‐SCI pain was evaluated by assessing sensitivity of the forepaws and skin of the back at the injury level. There was a nonsignificant trend for less sensitivity to tactile stimuli in transplanted compared with control animals, both for forepaws tested at 6 weeks after transplant/media injections (Fig. 2C left) and for the back tested at various, but particularly later, time points (Fig. 2C right). This indicates that transplants of hOM‐MSCs do not exacerbate pain and may even have some effect in preventing it.

### Gait Analysis

**Figure 3:**
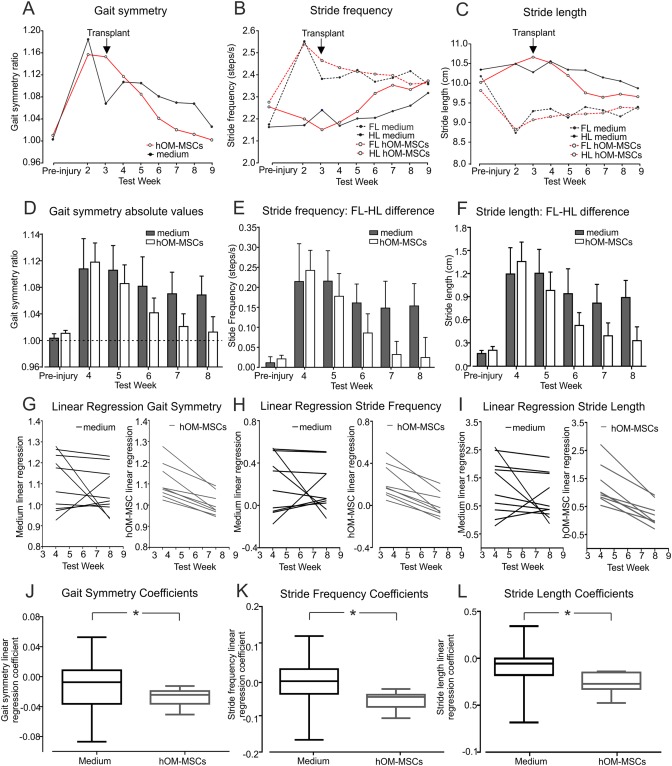
Gait parameters obtained by analysis of treadmill walking. (**A**–**F**) Changes in gait symmetry (ratio of forelimb (FL) to hindlimb (HL) steps), stride frequency and stride length. (**A**–**C**) Plots of mean ratios for pre‐injury and weekly post‐injury runs. (**D**–**F**) Bar graphs of mean ratios values (+ SEM) obtained pre‐injury and at 4, 5, 6, 7, and 8 weeks post‐injury. In (**B**) and (**C**), dotted lines represent forelimbs and solid lines hindlimbs. (**G**–**I**) Individual linear regression lines fitted through the values for gait symmetry (G), stride frequency difference (H) and stride length difference (I) at weeks 4–8 for all animals. (**J**–**L**) Linear regression coefficients were significantly more negative in the transplanted animals compared with media controls for gait symmetry (J), stride frequency (K) and stride length (L) revealing faster recovery of co‐ordinated stepping in hOM‐MSC transplanted group (*P < 0.05, all comparisons, Mann–Whitney test). A‐L, hOM‐MSC group *n* = 9, media group *n* = 10, at all time points. J, K, L, displayed as box and whisker plots. [Color figure can be viewed at wileyonlinelibrary.com]

Treadmill based analysis of gait showed progressive locomotor recovery following injury, with transplanted animals showing faster recovery of co‐ordinated stepping than control animals (Fig. 3). In normal animals, locomotion involves full co‐ordination between forelimbs and hindlimbs so that every forelimb step is accompanied by a hindlimb step. Therefore, before injury, the ratio of forelimb to hindlimb step frequency (termed gait symmetry) is 1 and deviation from this ratio indicates loss of co‐ordination. The plots in Fig. 3A,D show that this ratio changes after injury but then gradually recovers. However, the recovery appears to be faster and more complete in the transplanted animals compared with controls.

The plots in Fig. 3B,E showing changes in stride frequency (number of steps per unit time) and in Fig. 3C,F showing stride length, reveal the mechanism underlying these changes in co‐ordination. Following injury, there is an increase in the frequency of forelimb stepping, and since the animals are on a treadmill and therefore always run at the same speed, a corresponding reduction in stride length. In comparison, the frequency and stride length of the hindlimbs are initially unchanged after injury, so that the forelimbs and hindlimbs become dis‐coordinated. This may reflect a compensatory mechanism to provide greater stability and propulsion for locomotion when the hindlimbs are weakened. Furthermore, the gradual recovery of co‐ordination is explained by changes in the stride frequency of both the forelimbs and hindlimbs over time: the forelimbs progressively slow down while the hindlimbs speed up. Thus, closer co‐ordination is achieved by adaptation to a new common stride frequency, rather than normalisation of the stride frequency to that before the injury.

Because the plots in Fig. 3A–F suggest a more rapid improvement in co‐ordinated stepping in the transplanted animals, particularly in the first 5 weeks after cell injection, further analysis of gait symmetry, stride frequency and stride length parameters was performed using linear regression analysis. Individual linear regression lines were fitted through the values for each gait parameter at weeks 4 to 8 for all animals in the control and transplanted groups (Fig. 3G–I). The regression lines for all transplanted animals consistently show steep slopes corresponding to improved gait. In contrast, the slopes for control animals are much more variable and some even show an opposite slope indicating a deterioration of gait.

To test the statistical significance of these differences, linear regression coefficients were calculated (for gait symmetry, stride frequency and stride length parameters) using the slopes of the regression lines for each animal (Fig. 3G–I). Comparison of these linear regression coefficients for each parameter confirms a significantly faster improvement in the gait of transplanted animals compared with control (Fig. 3J–L, *P* < 0.05 for all, Mann–Whitney test).

### Electrophysiological Assessment

**Figure 4:**
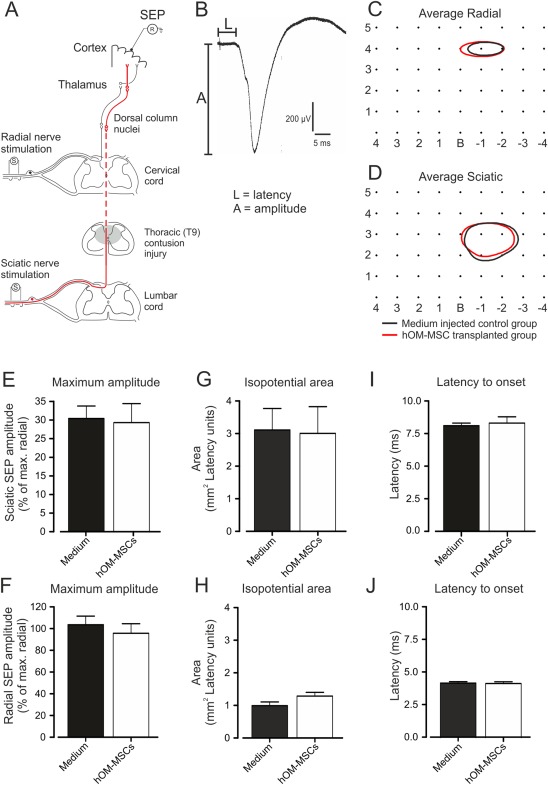
Electrophysiological assessment of transmission along the ascending dorsal column pathway. (**A**) Schematic diagram of the electrophysiological assessment showing position of stimulating and recording electrodes in relation to pathways originating in the forelimb (radial nerve afferents) and hindlimb (sciatic nerve afferents, red lines). (**B**) Example of SEP evoked by stimulation of the sciatic nerve and recorded from the sensorimotor cortex (average of 30 responses). Measurements of amplitude (A) and latency (L) at each recording location. (**C** and **D**) SEP isopotential contour plots for (**C**) radial nerve‐evoked potentials (80% of maximum) and (D) sciatic nerve‐evoked potentials (15% of maximum). Contours represent the means constructed from isopotential plots for hOM‐MSC transplanted (red; *n* = 13) and medium injected (black; *n* = 15) animal groups. Numbers on the isopotential grids represent distances in mm relative to Bregma (B). Comparison of SEP parameters. (**E** and **F**) Maximum amplitudes of sciatic‐evoked (**E**), radial‐evoked (F) and SEPs expressed as a percentage of the maximum radial SEP recorded in each animal (mean + SEM). (**G** and **H**) Mean areas of isopotential contour plots encompassing areas of sensorimotor cortex within which radial and sciatic SEP amplitudes were at least 80% and 15% of the maximal radial SEP amplitude recorded in each animal, respectively. (**I** and **J**) Latency to SEP onset for sciatic‐evoked (**I**) and radial‐evoked (J) potentials (mean + SEM). hOM‐MSC transplanted, *n* = 13; medium injected controls, *n* = 15. There were no significant differences in SEP amplitude, area or latency between groups (Student's unpaired *t* tests). [Color figure can be viewed at wileyonlinelibrary.com]

The effect of transplants on conduction along the dorsal column pathway and past the injury site (Fig. 4A) was assessed electrophysiologically at the end of the study by recording SEPs (Fig. 4B) evoked by the sciatic nerve. T9 contusion injuries of 150 kdynes reduce the SEP amplitude to about half of that seen in normal animals (data not shown). However, when recordings from transplanted and control animals were compared, there was no difference in the maximal amplitudes (Fig. 4E), distribution over the cortical surface (isopotential area, Fig. 4G) or onset latency (Fig. 4I) in the two groups. As would be expected, radial‐evoked SEPs, recorded as an internal control which were used to normalise the radial evoked SEPs, were also unchanged (Fig. 4F,H,J). This suggests that transplants have little effect on function in the injured dorsal column pathway, at least at the relatively chronic time point investigated.

### Injury Site Histology

**Figure 5:**
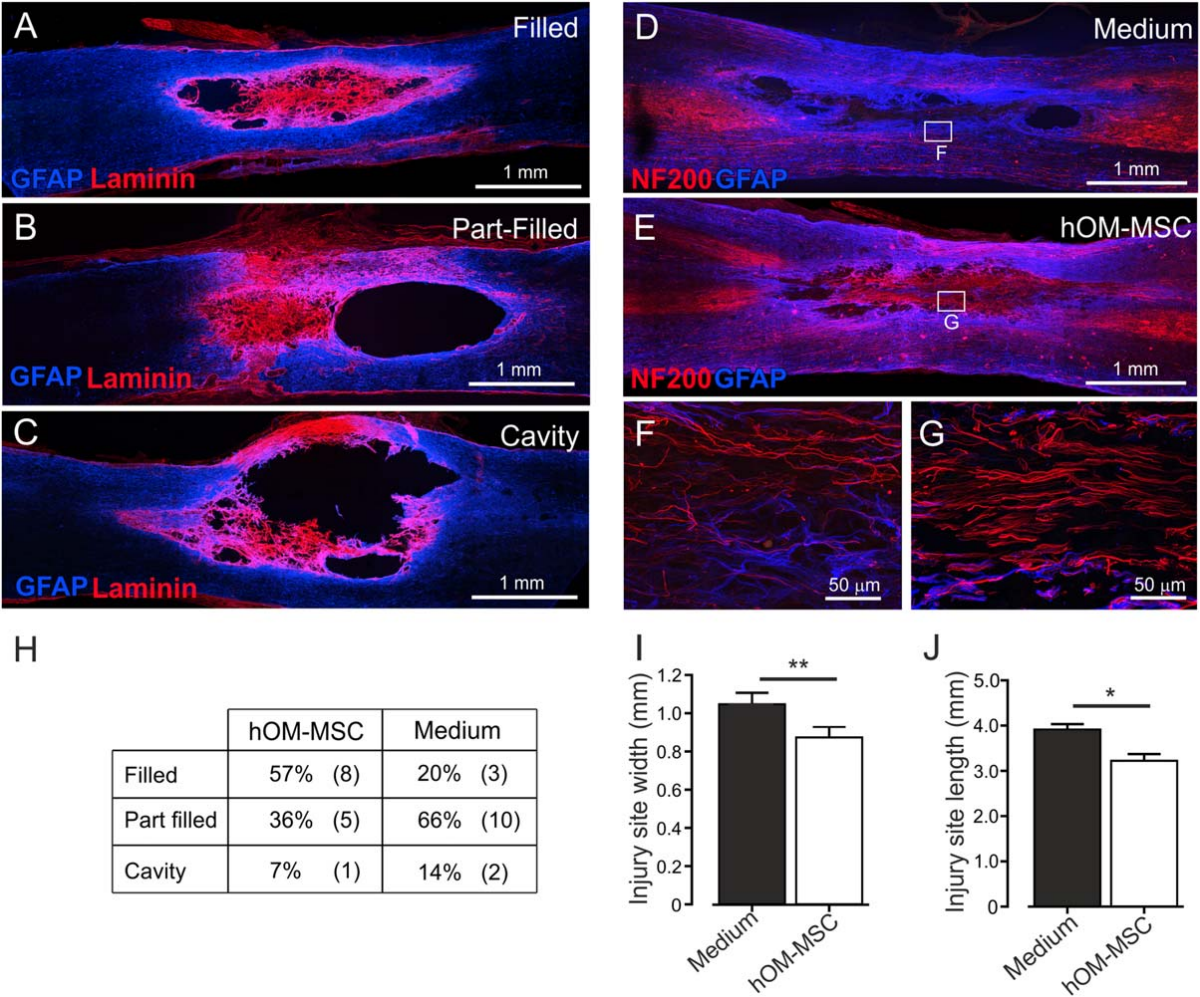
Characterisation of injury site animals following delayed transplantation into the contused spinal cord. (**A**–**C**) Confocal microscope images illustrating degrees of cavitation and extracellular matrix infilling of the injury site at 10 weeks post‐injury. Injury sites were classified as (A) filled (B) part‐filled or (C) cavity (GFAP = blue, Laminin = red). (**G**–**J)** Confocal images illustrating axonal regeneration within the cavity of (**D**) media injected or (**E**) hOM‐MSC transplanted animals (GFAP = blue, NF200 = red). **F** and G are higher magnification confocal images of the boxed areas in D and E, respectively. (H) Table showing incidence of each type of injury site in hOM‐MSC transplanted (*n* = 13) and medium injected animals (*n* = 15). (I) Injury site width and (J) injury site length (mean ± SEM). Injury sites were significantly less extensive in width and length in hOM‐MSC transplanted animals compared with media controls (Student's unpaired *t* test, **P < 0.01, *P < 0.05). [Color figure can be viewed at wileyonlinelibrary.com]

The histological appearance of the injury site in media injected and cell transplanted animals was classified as fully matrix filled, part matrix filled or an empty cavity (Fig. 5A–C). Transplanted animals tended to have more matrix filled cavities (57%) than media controls (20%) (Fig. 5H). Furthermore, the injury sites of transplanted animals were significantly less extensive in both length and width compared with media controls (Fig. 5I,J). In both groups of animals, where matrix filled or part filled the injury site it was rich in laminin and often contained numerous regenerating NF200 immunolabelled fibres (Fig. 5D–G).

### Peripheral Myelin Protein Zero (P0) Distribution

**Figure 6:**
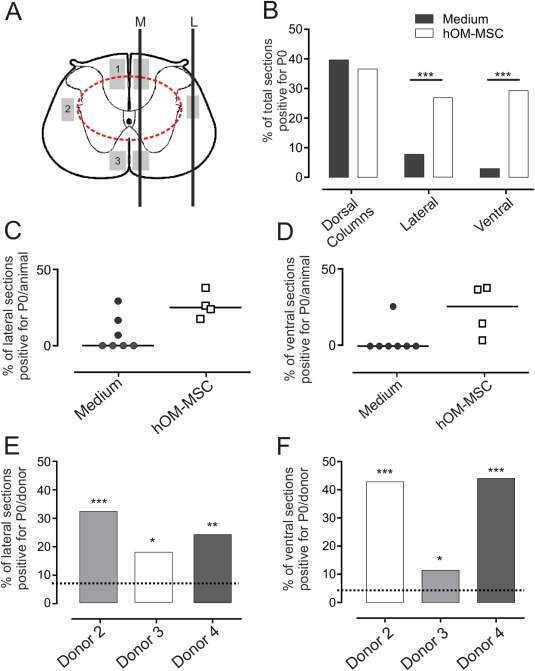
Peripheral myelin protein zero (P0) distribution within the injured spinal cord. (**A**) Schematic diagram of T9 transverse section through an injury site (represented by the dashed red line), illustrating regions where P0 immunolabelling was observed (grey shaded boxes in (1) dorsal columns, (2) lateral white matter and (3) ventral white matter). Black lines illustrate planes of sections passing through these regions at the midline (M) or lateral aspect (L) of the spinal cord. (**B**) Proportion of sections passing through each region depicted in (A) where P0 immunolabelled axons could be detected (percentage for all sections from all animals examined; hOM‐MSC, *n* = 4; media controls, *n* = 7). P0 immunolabelling occurred in significantly more sections from transplanted than control animals in lateral and ventral regions of the white matter (Non‐parametric fisher exact test of independence, ***P < 0.001), but in similar numbers of sections containing dorsal columns. (C, D) Scatter plots depicting percentage of lateral (**C**) and ventral (**D**) sections positive for P0 within each animal. The distribution between groups is significantly different. The majority of control animals have no P0 within lateral or ventral regions, compared with all transplanted animals which consistently have immunolabelling. (**E**) and (**F**) Bar graphs showing % sections with P0 immunolabelling from spinal cords of animals transplanted with each batch of donor cells. P0 remyelination is significantly increased in lateral (E) and ventral (**F**) regions, after transplantation with each donor cell batch compared with media injected controls (Control levels depicted by dashed lines. Non‐parametric fisher exact test of independence, *P < 0.05, **P < 0.01, ***P < 0.001). [Color figure can be viewed at wileyonlinelibrary.com]

Systematic observations of the distribution of P0 (a marker of peripheral type myelin) were made in the spared white matter surrounding the area of damage produced by the contusion where demyelination has been reported to occur in this model (James et al., 2011; Totoiu and Keirstead, 2005). These areas are divisible by anatomical location into the dorsal columns, the lateral funiculi and the ventral funiculi as depicted diagrammatically in Fig. 6A.

**Figure 7:**
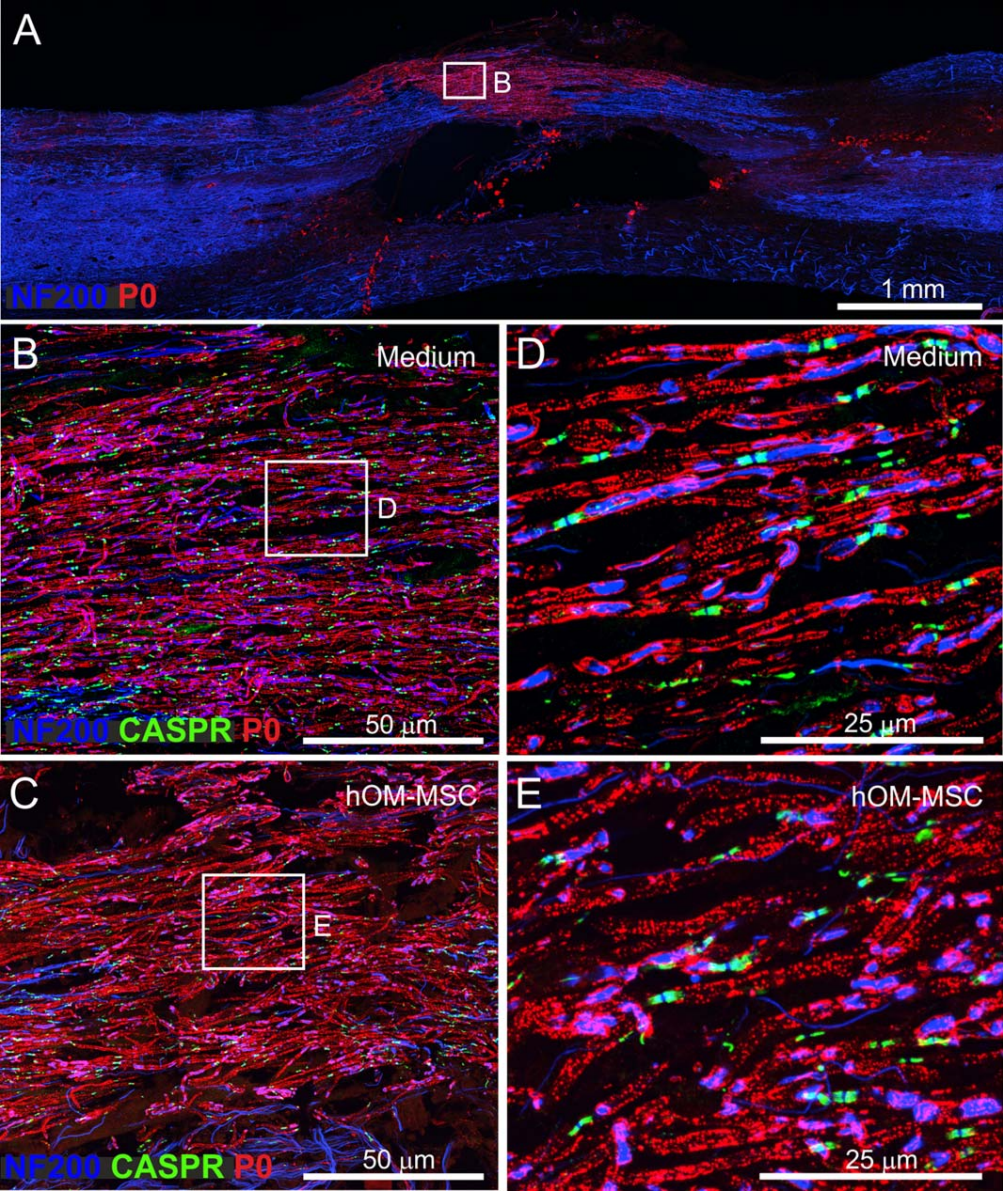
Peripheral myelin protein zero (P0) distribution within the dorsal columns of the injured spinal cord. (**A**) Confocal image showing typical region of dorsal columns at injury site center where P0 immunolabelling was seen in both medium injected and transplanted animals. The example is a control animal, 10 weeks post‐injury (P0 = red, NF200 = blue). (**B, C**) High power confocal images of P0 immunoreactivity within the dorsal columns of (B) medium injected and (C) transplanted animals, respectively. Boxed areas in (B) and (C) are shown at greater magnification in (**D**) and (**E**), respectively. (D) and (E) illustrate clear association of P0 with nerve fibres and Caspr (B–E, P0 = red, NF200 = blue, Caspr = green). [Color figure can be viewed at wileyonlinelibrary.com]

In the dorsal column white matter, P0 immunolabelling was detected in all of the animals investigated, both transplanted and control. The heaviest labelling was seen immediately dorsal to the contusion injury at the impact site (Fig. 7A) and this was common to all animals. In some animals, occasional P0 immunolabelled axons could also be seen scattered along the length of the section. High magnification confocal imaging of the main central area of P0 immunolabelling (Fig. 7B–E) revealed that nearly all neurofilament positive fibres in this area were associated with P0, suggesting extensive remyelination by Schwann cells forming peripheral type myelin. In addition, immunolabelling for the paranodal membrane protein Caspr, suggested the presence of normal nodal architecture. The appearance of these areas of dorsal column remyelination was similar in control and transplanted animals (cf, Fig. 7B,D and Fig. 7C,E). The remyelination was also similar in extent as the total number of sections in which P0 could be observed in the dorsal columns was closely similar in transplanted and control animals (Fig. 6B).

**Figure 8:**
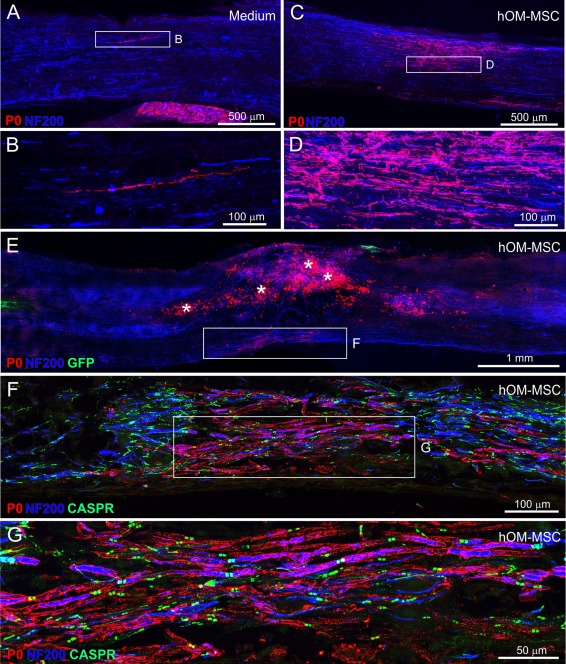
P0 distribution within lateral and ventral areas of the spinal cord. (**A**–**D**) Confocal images of P0 immunolabelling within lateral regions of medium injected (A, B) and hOM‐MSC transplanted (C, D) animals, respectively (P0 = red, NF200 = blue). (B) and (D) show high power images of boxed areas in (A) and (C), respectively. (**E**–**G**) Confocal images of P0 immunolabelling within ventral regions of hOM‐MSC transplanted animal (P0 = red, NF200 = blue, Caspr = green). (E) Low power view of the injury site. The asterisks indicate macrophages immunolabelled by cross‐reactivity with P0 antibody. Boxed area (E) contains P0 immunolabelling. High power image illustrating the distribution shown in (F). Boxed area in (F) is magnified in (**G**) showing association of P0 immunolabelling with nerve fibres and Caspr. [Color figure can be viewed at wileyonlinelibrary.com]

In contrast to the similar pattern of P0 immunolabelling in the dorsal columns, its distribution in the lateral and ventral areas of white matter (Fig. 6A) differed markedly between control and transplanted animals. In all 4 of the transplanted animals examined, a region of dense P0 immunolabelling was found in the white matter immediately lateral to the injury (Fig. 8C,D). This was typically restricted longitudinally to the impact site that is, an area adjacent to the center of the injury. In comparison, P0 immunolabelling in this area was not consistently seen in control animals and where detected, was very sparse. Semi‐quantitative analysis showed that, compared with transplanted animals, significantly fewer sections from control animals contained P0 immunolabelling in the lateral white mater (Fig. 6B). In fact, only 3 of the 7 control animals investigated showed any evidence of laterally located P0 (despite clear P0 immunolabelling in the dorsal columns of the same animals; Fig. 6C) and in 2 of these animals this amounted to only one or two isolated lengths of axon (Fig. 8A,B).

A similar picture was seen when the ventral white matter was examined. For all 4 transplanted animals P0 immunolabelling was observed in a region immediately below the injury in the ventral columns (Fig. 8E–G). The immunolabelling in this region was restricted longitudinally to the level of the impact in two animals but in the remaining two animals there was a more extensive longitudinal distribution, as in the dorsal columns. Semi‐quantitative analysis showed that, compared with transplanted animals, significantly fewer sections from control animals contained P0 immunolabelling in the ventral white mater (Fig. 6B). In fact, ventral P0 immunolabelling was seen in only 1 of the 7 media control animals and even in this one example, it was tightly restricted to a small region directly ventral to the injury center. In the remaining six control animals it was completely absent (Fig. 6D).

There were no obvious differences in the effect of the donor cell batches as each significantly increased P0 myelination within the ventral and lateral white matter when compared with control animals, (Fig. 6E,F).

### hOM‐MSC Survival and Distribution

**Figure 9:**
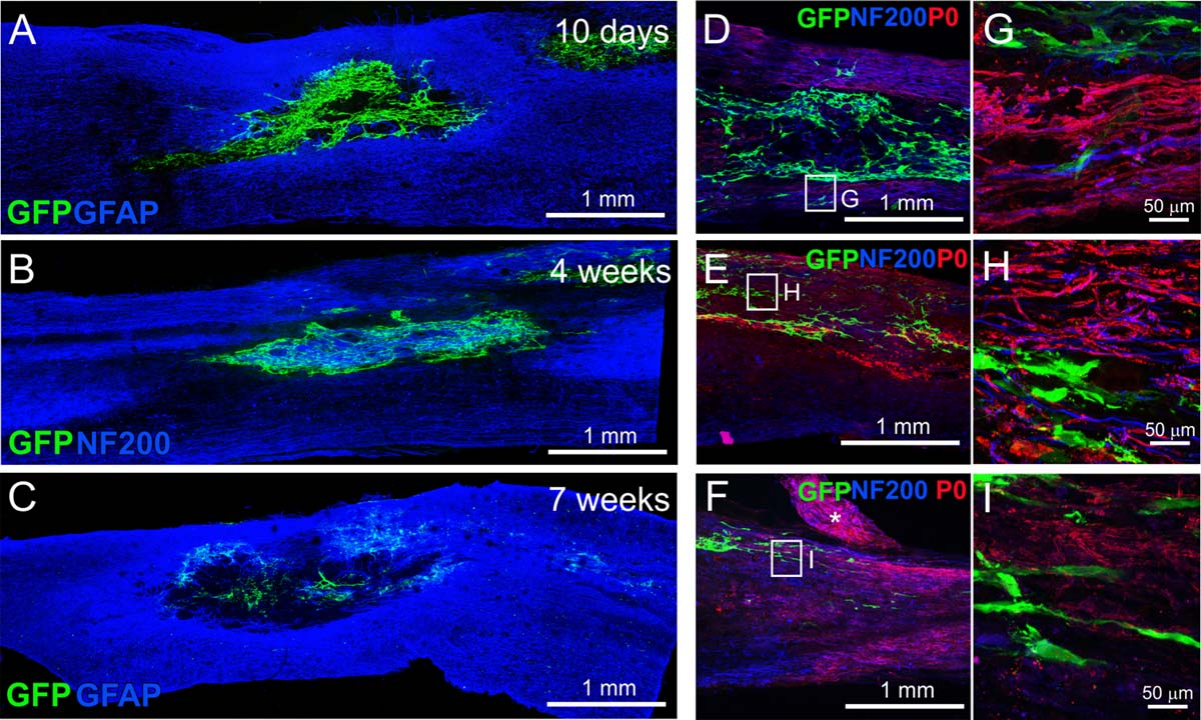
**hOM‐MSC survival and association with P0 distribution**. Confocal images showing survival of transplanted cells at and around the injury epicenter at different time points post‐transplantation: (**A**) 10 days, (**B**) 4 weeks and (**C**) 7 weeks (GFAP = blue, GFP expressing hOM‐MSCs = green (in A and C) and NF200 = blue, GFP expressing hOM‐MSCs = green (in B)). (**D**–**F**) Confocal images of GFP labelled hOM‐MSCs, NF200 axons and P0 immunolabelling found within dorsal columns (D), lateral funiculi (E) and ventral funiculi (F) in hOM‐MSC transplanted animal at 4 weeks (P0 = red, NF200 = blue, GFP = green). The boxed areas in (D–F) are shown at higher magnification in (**G**–**I**). The asterisk in (E) indicates a P0 immunolabelled dorsal root. [Color figure can be viewed at wileyonlinelibrary.com]

hOM‐MSC distribution and survival was assessed at 10 days, 4 weeks and 7 weeks post‐transplantation. At the early time points hOM‐MSCs were numerous and distributed throughout the injury site (Fig. 9A,B). However, by the end point of the main investigation (7 weeks) although GFP labelled cells were detected in all 15 transplanted animals, there were far fewer surviving cells (Fig. 9C). The distribution of hOM‐MSCs was assessed in relation to P0 and NF200 immunolabelling of axons at 4 weeks post‐transplantation when cells were still numerous, as well as in the 7 week survival animals. There was no association of GFP labelled hOM‐MSC profiles with either P0 remyelinated fibres or NF200 immunolabelled axons in the dorsal columns (Fig. 9D,G), lateral funiculi (Fig. 9E,H) or within the ventral funiculi (Fig. 9F,I). This suggests that hOM‐MSCs were not directly responsible for the P0 remyelination of spared fibres in these regions.

## Discussion

### Remyelination at the Injury Site

Demyelination is a potential therapeutic target for improving outcome after SCI (Plemel et al., 2014) because loss of the myelin sheath around spared axons is a pathological feature of traumatic injuries. Demyelination has consistently been reported in animal models of contusion injuries (Blight and Young, 1989; Bresnahan, 1978; Jeffery and Blakemore, 1999) and although there is also evidence from animal studies that demyelinated axons at the site of injury may be remyelinated by endogenous cells (Beattie et al., 1997; Biernaskie et al., 2007; Bresnahan, 1978; Brook et al., 1998; Bunge et al., 1993; Buss et al., 2007; Guest et al., 2005) this process is generally considered to be incomplete (Plemel et al., 2014). There are also reports of demyelination in clinical cases of SCI (Bunge et al., 1993; Guest et al., 2005; Kakulas, 1999; Norenberg et al., 2004), although the incidence and persistence is more difficult to assess because the evidence is generally obtained from post‐mortem material in which persistently demyelinated axons may have degenerated (Bunge et al., 1993).

Endogenous repair of demyelinated axons is known to be mediated in part by SCs and studies in animal models have consistently shown the presence of either SCs or P0 myelination within the spared rim of dorsal column white matter above the injury site (Beattie et al., 1997; Biernaskie et al., 2007; McTigue et al., 2006; Takami et al., 2002). Furthermore, this remyelination has been shown to lead to electrophysiologically demonstrable improvements in conduction along the dorsal columns (Blight and Young, 1989). In this study P0 immunolabelling was consistently seen in the dorsal columns and was of similar extent in both hOM‐MSC transplanted and control animals. This suggests efficient endogenous remyelination of dorsal column fibres by the end point of the study (10 weeks after injury) in both control and transplanted animals and this may explain why electrophysiological assessment of dorsal column conduction (SEP recordings) did not reveal a difference between the groups. In contrast to this general dorsal column labelling, in lateral and ventral regions of the white matter, P0 immunolabelling was only consistently observed in transplanted animals. In media injected animals P0 immunolabelling in these regions was usually absent or, in the minority of animals in which it was seen, involved only a few fibres. Consistent with this, there is only one previous report of P0 in regions lateral and ventral to a contusion injury and this was at a chronic time point (13 weeks post‐injury) (McTigue et al., 2006). P0 immunolabelled fibres in lateral and ventral white matter also immunolabelled for the paranodal protein Caspr supporting the premise that remyelinated fibres in these regions should be capable of supporting impulse conduction.

### Mechanism of Remyelination

Although P0 immunolabelling was more widely distributed in transplanted animals than controls, there was no evidence that the transplanted cells themselves formed myelin sheaths. Careful examination of all sections in which P0 immunolabelling associated with fibres was seen, did not show any examples of co‐localisation with GFP labelled hOM‐MSCs. This suggests that the transplanted cells do not themselves directly participate in the remyelination. This is consistent with the literature that transplanted MSCs do not differentiate *in vivo* but instead modulate the injury microenvironment (Zhang et al., 2015). In addition, when investigated *in vitro* these cells did not directly myelinate neurites in myelinating co‐cultures (Lindsay et al., 2013), rather, they were found to facilitate the formation of myelin by production of secreted factors.

Repair of myelin by endogenous cells is a well‐established phenomenon in the dorsal columns of the spinal cord and non‐myelinating and/or de‐differentiated SCs migrating from the dorsal roots are generally considered to contribute to this (Beattie et al., 1997; Duncan and Hoffman, 1997; Nagoshi et al., 2011). However, there is also evidence that precursor cells present within the CNS can give rise to remyelinating SCs (Akiyama et al., 2001; Blakemore, 2005; Keirstead et al., 1999; Zawadzka et al., 2010). At present the relative contribution of each of these potential sources of SCs is uncertain. We assume that the cells contributing to the peripheral type myelin seen in the lateral and ventral white matter of transplanted animals in this study have a similar origin. However, it is possible that there are regional differences. For example, remyelination in lateral regions (farther from spinal roots) might depend more on differentiation of precursor cells, while SCs in the ventral roots might contribute to remyelination in the ventral white matter.

The more extensive pattern of P0 immunolabelling seen in transplanted animals suggests that hOM‐MSCs facilitate Schwann cell remyelination over a wider area of white matter than would normally occur in the absence of cells. Transplant‐facilitated recruitment of endogenous SCs has been described previously (Biernaskie et al., 2007; Hill et al., 2006; Ramer et al., 2004; Takami et al., 2002), but only within the transplanted injury site and/or dorsal columns. The mechanism may involve chemotaxis induced by growth factors or cytokines produced by the transplanted cells (Nagoshi et al., 2011). hOM‐MSCs secrete a variety of factors including NGF (Lindsay et al., 2013) which is known to promote axonal susceptibility to Schwann cell myelination (Chan et al., 2004).

Although both SCs and OPCs participate in endogenous remyelination at a site of spinal cord injury, remyelination by SCs precedes that by OPCs (Totoiu and Keirstead, 2005) and preferentially targets large diameter axons. This early repair process is thought to be important for the rapid restoration of saltatory conduction and neuroprotection of demyelinated spared axons in the early stages following injury (Powers et al., 2013). Because hOM‐MSCs promote OPC differentiation and proliferation, and also promote CNS myelination *in vitro* (Lindsay et al., 2013), remyelination by oligodendrocytes may also have been facilitated in the transplanted animals studied here. Further investigation using electron microscopic or teased fibre approaches would be necessary to explore this possibility.

### Functional Outcome Measures

Dorsal column function did not differ in transplanted and control animals when assessed electrophysiologically at the end point of the study. However, this is probably explained by the highly efficient endogenous remyelination that occurs in the dorsal columns, as previously discussed. Gross assessment of locomotor recovery (BBB open field score) and posture (distribution of weight bearing) also did not differ between control and transplanted animals suggesting that any recovery of function resulting from the more extensive transplant‐mediated remyelination is relatively modest. However, more sophisticated and sensitive analysis of gait revealed a significantly more rapid recovery of the co‐ordination between forelimbs and hindlimbs during walking on the DigiGait treadmill in transplanted animals. The rate of improvement in gait co‐ordination was significantly greater in transplanted than control animals when analyzed using linear regression analysis (Matthews, 1993; Vossoughi et al., 2012). Co‐ordinated stepping primarily involves descending and long propriospinal fibres in lateral and ventral parts of the spinal cord (Gorska et al., 1996; Loy et al., 2002; Reed et al., 2009; Zorner et al., 2010), regions where peripheral myelination was enhanced in transplanted animals. It is therefore tempting to suggest that the greater improvement in gait seen in transplanted animals is associated with the more extensive remyelination. Injury site cavitation was also reduced in the transplanted animals and this might have contributed to the improved gait.

### Comparison with Previous Cell Transplant Studies

This is the first report to assess the repair potential of purified transplants of MSCs from the human olfactory mucosa: nonetheless there have been numerous studies using this tissue as a source of cells. Where nonpurified cells have been used (Chhabra et al., 2009; Feron et al., 2005; Granger et al., 2012; Lima et al., 2010, 2006), it is probable that hOM‐MSCs were present since they proliferate quickly and express surface markers which overlap with those expressed by fibroblast‐like cells (Delorme et al., 2010; Lindsay et al., 2013). Of particular interest is a recent study in which dogs with naturally occurring chronic SCI were treated with autologous cells derived from olfactory mucosa (Granger et al., 2012). The functional outcome reported was very similar to this study in that although long tract function assessed electrophysiologically did not improve, gait analysis showed more co‐ordinated stepping. However, the mechanisms underlying these improvements could not be investigated (Granger et al., 2012).

Although MSCs have been widely investigated in the field of SCI their source has, until now, been limited to bone marrow (BM‐MSCs). There are no reports of BM‐MSCs leading to improved remyelination as we show here for hOM‐MSCs, and our own *in vitro* investigations have shown that conditioned media from BM‐MSCs does not have the same facilitatory effect on CNS myelination (Lindsay et al., 2013). Although there are reports of functional improvements when transplanted acutely after injury (Ankeny et al., 2004; Himes et al., 2006; Hofstetter et al., 2002; Neuhuber et al., 2005; Torres‐Espin et al., 2014), minimal improvements are seen when transplantation is delayed to subacute or chronic time points (Nandoe Tewarie et al., 2009; Tetzlaff et al., 2011; Torres‐Espin et al., 2014; Yoshihara et al., 2006).

Alternative strategies for the promotion of remyelination by cell therapy include transplanting cells which directly participate in remyelination [reviewed by (Plemel et al., 2014; Tetzlaff et al., 2011)]. For example, recent investigations have shown that embryonic stem cell derived OPCs (Sharp et al., 2010) and SCs, including cells derived from skin precursors (Sparling et al., 2015) enhanced remyelination and functional recovery in rodent contusion injury models, although these studies used subacute (7 days) and acute transplantation, respectively and mechanisms other than remyelination may have contributed to functional recovery.

The results reported here suggest that hOM‐MSCs have the potential to enhance endogenous remyelination after traumatic SCI. Because persistent demyelination may be a pathological feature of at least some clinical injuries this may be therapeutically beneficial in accelerating functional recovery and/or providing neuroprotection of spared axons.
